# Fluoroquinolones versus β-Lactam/β-Lactamase Inhibitors in Outpatients with Chronic Obstructive Pulmonary Disease and Pneumonia: A Nationwide Population-Based Study

**DOI:** 10.1371/journal.pone.0136232

**Published:** 2015-08-25

**Authors:** Kuan-Yin Lin, Chi-Chuan Wang, Chia-Hui Lin, Wang-Huei Sheng, Shan-Chwen Chang

**Affiliations:** 1 Center for Drug Evaluation, Taipei, Taiwan; 2 School of Pharmacy, National Taiwan University, Taipei, Taiwan; 3 Department of Pharmacy, National Taiwan University Hospital, Taipei, Taiwan; 4 Departments of Internal Medicine, National Taiwan University Hospital and National Taiwan University College of Medicine, Taipei, Taiwan; University of Dundee, UNITED KINGDOM

## Abstract

**Background:**

Studies on the association between antibiotic treatment and outcomes in outpatients with chronic obstructive pulmonary disease (COPD) and pneumonia are scarce. This study aimed to evaluate the effectiveness of fluoroquinolones and β-lactam/β-lactamase inhibitors for pneumonia in COPD outpatients.

**Methods:**

We conducted a retrospective cohort study and identified 4,851 episodes of pneumonia among COPD outpatients treated with fluoroquinolones or β-lactam/β-lactamase inhibitors from the Taiwan National Health Insurance Research Database during 2002–2011. Using the propensity score analysis, 1,296 pairs of episodes were matched for the demographic and clinical characteristics. The primary outcome was pneumonia/empyema-related hospitalization or emergency department (ED) visits, and the secondary outcomes were treatment failure, all-cause mortality and medical costs within 30 days.

**Results:**

Compared with episodes treated with β-lactam/β-lactamase inhibitors, episodes treated with fluoroquinolones had similar clinical outcomes. The rates of pneumonia/empyema-related hospitalization or ED visits were 3.9% and 3.5% in the fluoroquinolone and β-lactam/β-lactamase inhibitor groups, respectively (adjusted hazard ratio [aHR], 1.11; 95% confidence interval [CI], 0.74–1.66). The percentage of treatment failure and all-cause mortality were 28.2% versus 31.3% (adjusted odds ratio, 0.86; 95% CI, 0.73–1.02) and 0.5% versus 0.4% (aHR, 1.40; 95% CI, 0.45–4.41) in the fluoroquinolone and β-lactam/β-lactamase inhibitor groups, respectively. The medical expenditures, including total medical costs (528 versus 455 US dollars) and pneumonia-related costs (202 vs. 155 USD) were also balanced between the two treatment groups (both *P* >0.05).

**Conclusions:**

For pneumonia in COPD outpatients, fluoroquinolones were associated with similar clinical outcomes and medical expenditures compared with β-lactam/β-lactamase inhibitors.

## Introduction

Chronic obstructive pulmonary disease (COPD) is a major cause of chronic morbidity and mortality worldwide [1], and projected to become the fourth leading cause of death and the seventh cause of the global burden of disease by 2030 [2]. The burden of COPD in Asia is even greater than that in the developed Western countries [3]. COPD patients more frequently have community-acquired pneumonia (CAP), and COPD accounts for 15% to 42% of the comorbidity of CAP [4]. The increased risk of pneumonia in COPD patients results from parenchymal destruction, chronic inflammation, and treatment with inhaled corticosteroids [5,6].

The choice of antibiotic treatment for pneumonia among the COPD population is different from other populations in consideration of the diverse microbiological pattern and bacterial resistance. *Streptococcus pneumoniae* is the most common causative organism in both COPD and non-COPD patients, whereas *Haemophilus influenzae* and *Pseudomonas aeruginosa* infections are more frequent in COPD patients. In addition, the emergence of penicillin-resistant *S*. *pneumoniae* (PRSP) and β-lactamase-producing *H*. *influenzae* may also be a concern [4,7]. Current Infectious Diseases Society of America/American Thoracic Society (IDSA/ATS) and European Society of Clinical Microbiology and Infectious Diseases (ESCMID) treatment guidelines for pneumonia recommend a respiratory fluoroquinolone or combination of a β-lactam and a macrolide in outpatients with comorbidities or risks for acquiring resistant microorganisms [7,8].

Several clinical trials and meta-analyses have compared the effectiveness of fluoroquinolones and β-lactams with or without macrolides in acute exacerbation of COPD (AECOPD), and some of them showed significant bacterial eradication and improved long-term outcomes in patients receiving fluoroquinolones [9,10]. These findings were supported by the evidence that fluoroquinolones had a broader spectrum and lower resistant rate [11]. Moreover, fluoroquinolones offer the excellent penetration into the bronchial mucosa and have the pharmacokinetic profile allowing once-daily oral dosing regimen [11]. Despite these advantages and widespread use of fluoroquinolones, there is limited information on antibiotic choice of pneumonia in COPD patients [12]. In this study, we aimed to evaluate the clinical outcomes and medical expenditures of fluoroquinolones and β-lactam/β-lactamase inhibitors for pneumonia among COPD outpatients using the nationwide population-based cohort.

## Methods

### Study population

The source population comprised over 99% of the entire population in Taiwan (23 million inhabitants) and the database was from the National Health Insurance Research Database (NHIRD), which was established by the Bureau of NHI and the National Health Research Institute. To ensure all beneficiaries’ privacy and confidentiality, the NHIRD was provided to researchers only upon ethical approval. This study was approved by the Research Ethics Committee of National Taiwan University Hospital (registration number, NTUH-201501012W).

This study used three subsets of the NHIRD, including Longitudinal Health Insurance Database (LHID) 2000, 2005, and 2010, and each of the LHID consisted of 1 million individuals randomly selected from registry of beneficiaries enrolled in 2000, 2005 or 2010, respectively [13]. These databases contained information on all medical claims, including diagnoses, prescriptions, outpatient visits, hospital admissions, and healthcare procedures. Diagnoses were defined according to the International Classification of Diseases, Ninth Revision, Clinical Modification (ICD-9-CM).

### Study design

We conducted a retrospective cohort study during 2002 to 2011. Patients were included if they were aged 20 years or older, with the comorbidity of COPD and experiencing at least one pneumonia episode. In order to increase the validity of the diagnosis of COPD, only those patients with at least 2 outpatient diagnoses more than 30 days apart or 1 inpatient diagnosis of COPD (ICD-9-CM 490–492 and 496) were identified as COPD patients. A pneumonia episode was defined as the primary diagnosis of pneumonia in outpatient visits (ICD-9-CM 481–483, 484.8, 485–486, and 487.0) plus a confirmatory diagnostic procedure, such as either of chest X-ray, complete blood count, sputum smear or culture, and blood culture. The day of pneumonia diagnosed was defined as the index date. These included patients had to initiate oral fluoroquinolones (gemifloxacin, moxifloxacin, or levofloxacin) or oral β-lactam/β-lactamase inhibitors with or without macrolides (erythromycin, azithromycin or clarithromycin) on the index date, and had continued antibiotics for at least 5 consecutive days. We excluded patients who were prescribed other concomitant antibiotics on the index date and any antibiotics in previous 15 days, hospitalized in previous 30 days, pregnant in the past year, and major immunosuppressed status (patients with HIV/AIDS or transplantation, receiving cancer chemotherapy, immunoglobulin and immune-targeted therapy in the past year).

We collected several covariates, including demographics, baseline comorbidities, recent medication, and utilization of healthcare services, which were recorded within one year before the index date. Baseline comorbidities were classified into respiratory diseases, cardiovascular diseases, peptic ulcer disease, diabetes mellitus, malignancies, chronic liver and renal diseases. Recent medication included any antibiotics, disease-modifying antirheumatic drugs, and long-term oral corticosteroids for at least consecutive 30 days within one year. The utilization of healthcare services were categorized into four groups: COPD-related outpatient visits, COPD-related hospitalizations or emergency department (ED) visits, respiratory disease-related outpatient visits, and respiratory disease-related hospitalizations or ED visits.

### Outcome measures

The primary outcome was pneumonia or empyema-related hospitalizations or ED visits. Hospitalizations or ED visits within the first 72 hours following the index date didn’t meet the primary outcome measure because the effect of antibiotics on pneumonia should be evaluated in 72 hours later [14]. The secondary outcomes included treatment failure, all-cause mortality, and medical costs. The duration of follow-up was 30 days after the index date. The definition of treatment failure consisted of the following: (1) prolonged antibiotic treatment for more than 14 days; (2) change or add another antibiotic different from study medication; (3) switch from oral antibiotics to intravenous antibiotics. We assessed medical expenditures by total medical costs and pneumonia-related costs, which were the sum of outpatient and inpatient medical costs, and the costs were presented in US Dollars (USDs). In consideration of possible masking of active tuberculosis by fluoroquinolones, we assessed tuberculosis diagnosed after the treatment of pneumonia. After excluding the episodes diagnosed as tuberculosis during the preceding one-year period, the episodes of newly diagnosed tuberculosis were identified in 6 months after the index date.

### Statistical analysis

In the description of demographics and clinical characteristics, categorical variables were compared with a chi-square test, and continuous variables were compared using the Student’s *t*-test. All tests were two-tailed and *P* < 0.05 was considered statistically significant. Kaplan–Meier estimates of time to hospitalization/ED visits and 30-day mortality were calculated. We used Cox-proportional hazard models to compare the rates of hospitalization/ED visits and all-cause mortality between the treatment groups, assessed the likelihood of treatment failure by a logistic regression model, and compared medical expenditures by the Student’s *t*-test. Ninety-five percent confidence intervals (CIs) of hazard ratios (HRs) or odds ratios (ORs) were computed.

In an attempt to balance the differences of covariates between episodes treated with fluoroquinolones and β-lactam/β-lactamase inhibitors, we performed the propensity score matching analysis [15]. Based on the estimated propensity score, greedy match was used to select episodes into the subsequent analysis. Unadjusted and propensity score-adjusted models were both evaluated. Statistical analyses were performed using SAS software version 9.3 (SAS Institute Inc., Cary, NC, USA).

## Results

**Fig 1:**
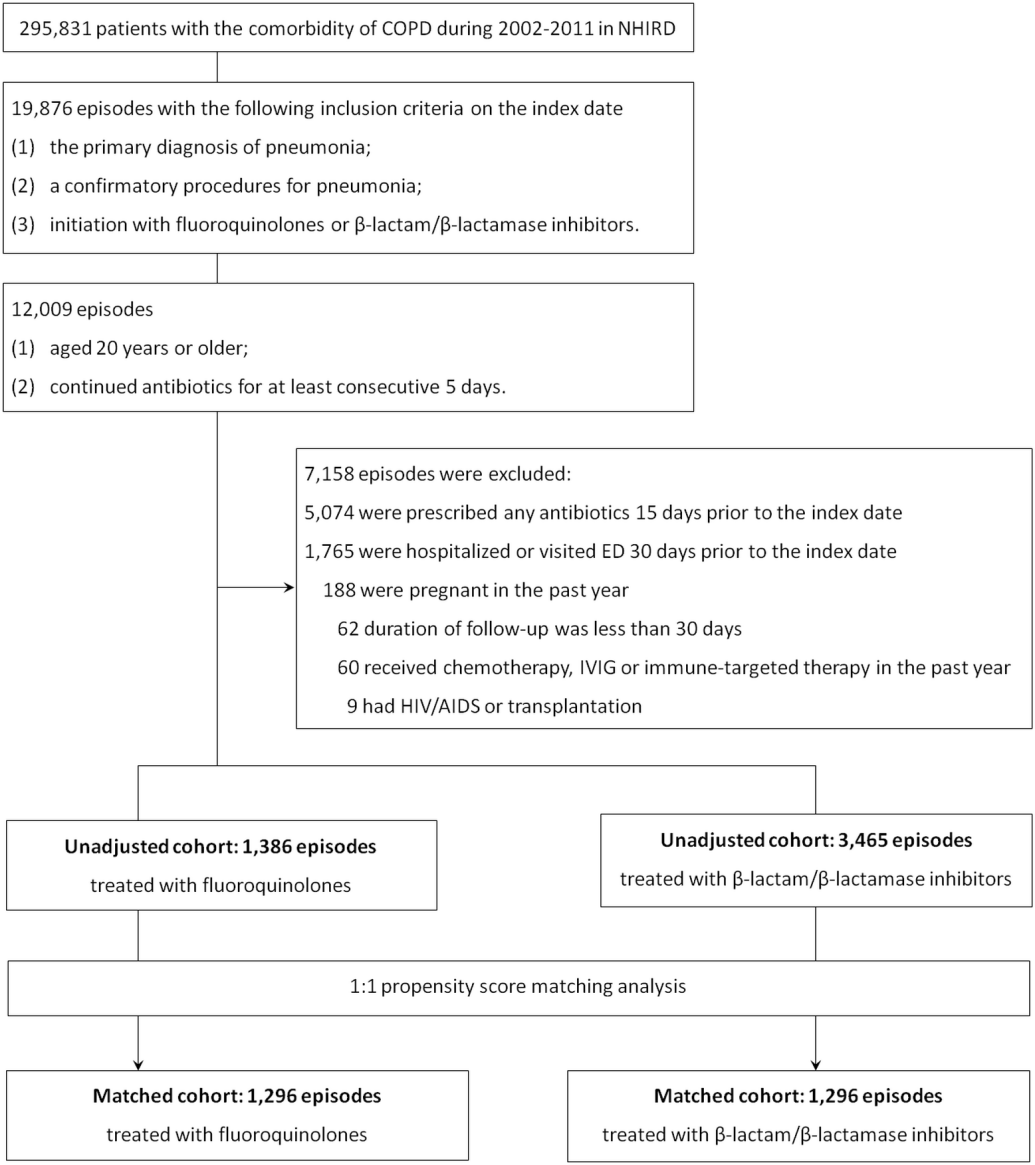
Flow diagram of patient selection. **Abbreviations:** COPD, chronic obstructive pulmonary disease; ED, emergency department; IVIG, intravenous immunoglobulin; NHIRD, National Health Insurance Research Database.

A total of 4,851 episodes of pneumonia among COPD patients were included in the analyses. Of them, 1,386 episodes were treated with fluoroquinolones and 3,465 episodes were treated with β-lactam/β-lactamase inhibitors plus macrolides or not, respectively. After propensity score matching, 1,296 matched pairs of episodes were matched with a one-to-one ratio (Fig 1). The demographics and clinical characteristics of episodes in the unadjusted cohorts treated with fluoroquinolones and β-lactam/β-lactamase inhibitors are listed in Table 1. Compared with episodes treated with β-lactam/β-lactamase inhibitors, episodes treated with fluoroquinolones were younger (66.0 years versus 67.3 years, *P* = 0.01), less likely to be male (54.9% versus 61.1%, *P*<0.01), and had a lower proportion of cerebrovascular diseases (17.2% vs. 21.2%, *P*<0.01). In both fluoroquinolone and β-lactam/β-lactamase inhibitor groups, there were over 70% and 30% of patients exposed to any antibiotics in the past 1 year and 3 months. Long-term oral corticosteroids were prescribed in about one-third of patients in the previous year. The rate of hospitalization or ED visits due to acute exacerbation of COPD or pneumonia in the past year was 5% of our study patients. In attempt to balance the imbalance of covariates between two treatment groups, the propensity score matching analysis was performed. The demographics and clinical characteristics of adjusted cohorts, also listed in Table 1, were all balanced.

**Table 1:** Demographics and clinical characteristics of episodes treated with fluoroquinolones and β-lactam/β-lactamase inhibitors in unadjusted cohort and matched cohort.

	Unadjusted cohort			Matched cohort		
	Fluoroquinolones(N = 1386)	β-lactam/β-lactamase inhibitors (N = 3465)	*P*	Fluoroquinolones(N = 1296)	β-lactam/β-lactamase inhibitors (N = 1296)	*P*
Age, mean ± SD (years)	66.0 ± 16.4	67.3 ± 15.7	0.01	65.8 ± 16.4	65.7 ± 15.9	0.90
Male, n (%)	761 (54.9)	2116 (61.1)	<0.01	710 (54.8)	686 (52.9)	0.34
**Comorbidity, n (%)**						
Respiratory disease						
Bronchitis and bronchiolitis	592 (42.7)	1508 (43.5)	0.61	556 (42.9)	544 (42.0)	0.63
Previous pneumonia	424 (30.6)	1035 (29.9)	0.62	361 (27.9)	366 (28.2)	0.83
Asthma	263 (19.0)	623 (18.0)	0.42	238 (18.4)	226 (17.4)	0.54
Tuberculosis	39 (2.8)	137 (4.0)	0.06	36 (2.8)	41 (3.2)	0.56
Chronic sinusitis	37 (2.7)	79 (2.3)	0.42	30 (2.3)	33 (2.5)	0.70
Pleurisy	36 (2.6)	81 (2.3)	0.59	34 (2.6)	33 (2.5)	0.90
Other respiratory diseases^a^	170 (12.3)	426 (12.3)	0.97	153 (11.8)	146 (11.3)	0.67
Cardiovascular disease						
Hypertension	510 (36.8)	1345 (38.8)	0.19	476 (36.7)	485 (37.4)	0.71
Coronary artery disease	311 (22.4)	715 (20.6)	0.16	280 (21.6)	273 (21.1)	0.74
Heart failure or cardiomyopathy	140 (10.1)	350 (10.1)	0.99	130 (10.0)	129 (10.0)	0.95
Cerebrovascular disease	238 (17.2)	734 (21.2)	<0.01	216 (16.7)	222 (17.1)	0.75
Peripheral arterial disease	24 (1.7)	55 (1.6)	0.72	23 (1.8)	16 (1.2)	0.26
Peptic ulcer disease	295 (21.3)	786 (22.7)	0.29	272 (21.0)	302 (23.3)	0.16
Diabetes mellitus	264 (19.0)	678 (19.6)	0.68	249 (19.2)	242 (18.7)	0.73
Malignancy	221 (15.9)	493 (14.2)	0.13	201 (15.5)	214 (16.5)	0.49
Chronic liver disease	163 (11.8)	411 (11.9)	0.92	158 (12.2)	160 (12.3)	0.90
Chronic renal disease	99 (7.1)	213 (6.1)	0.20	94 (7.3)	86 (6.6)	0.54
**Medication in the past year, n (%)**						
Any antibiotics	1027 (74.1)	2514 (72.6)	0.27	948 (73.1)	940 (72.5)	0.72
Any antibiotics within 3 month	494 (35.6)	1196 (34.5)	0.46	450 (34.7)	423 (32.6)	0.26
DMARD	14 (1.0)	25 (0.7)	0.31	9 (0.7)	15 (1.2)	0.22
Long-term corticosteroids	455 (32.8)	1110 (32.0)	0.59	421 (32.4)	414 (31.9)	0.77
**Utilization of healthcare services in the past year, n (%)**						
COPD-related outpatient visits	293 (21.1)	756 (21.8)	0.60	268 (20.7)	260 (20.1)	0.70
COPD-related hospitalization or ED visits	14 (1.0)	24 (0.7)	0.26	14 (1.1)	13 (1.0)	0.85
Respiratory disease-related outpatient visits^b^	309 (22.3)	740 (21.4)	0.47	259 (20.0)	264 (20.4)	0.81
Respiratory disease-related hospitalization or ED visits	48 (3.5)	148 (4.3)	0.20	42 (3.2)	34 (2.6)	0.35

**Abbreviations:** COPD, chronic obstructive pulmonary disease; DMARD, disease-modifying antirheumatic drugs; ED, emergency department; SD, standard deviation.

^a^Other respiratory diseases included dysplasia of lung, cystic fibrosis, pulmonary fibrosis, pneumoconiosis, pneumothorax, pulmonary congestion, empyema, and lung abscess.

^b^Respiratory diseases included pneumonia and asthma.

**Fig 2:**
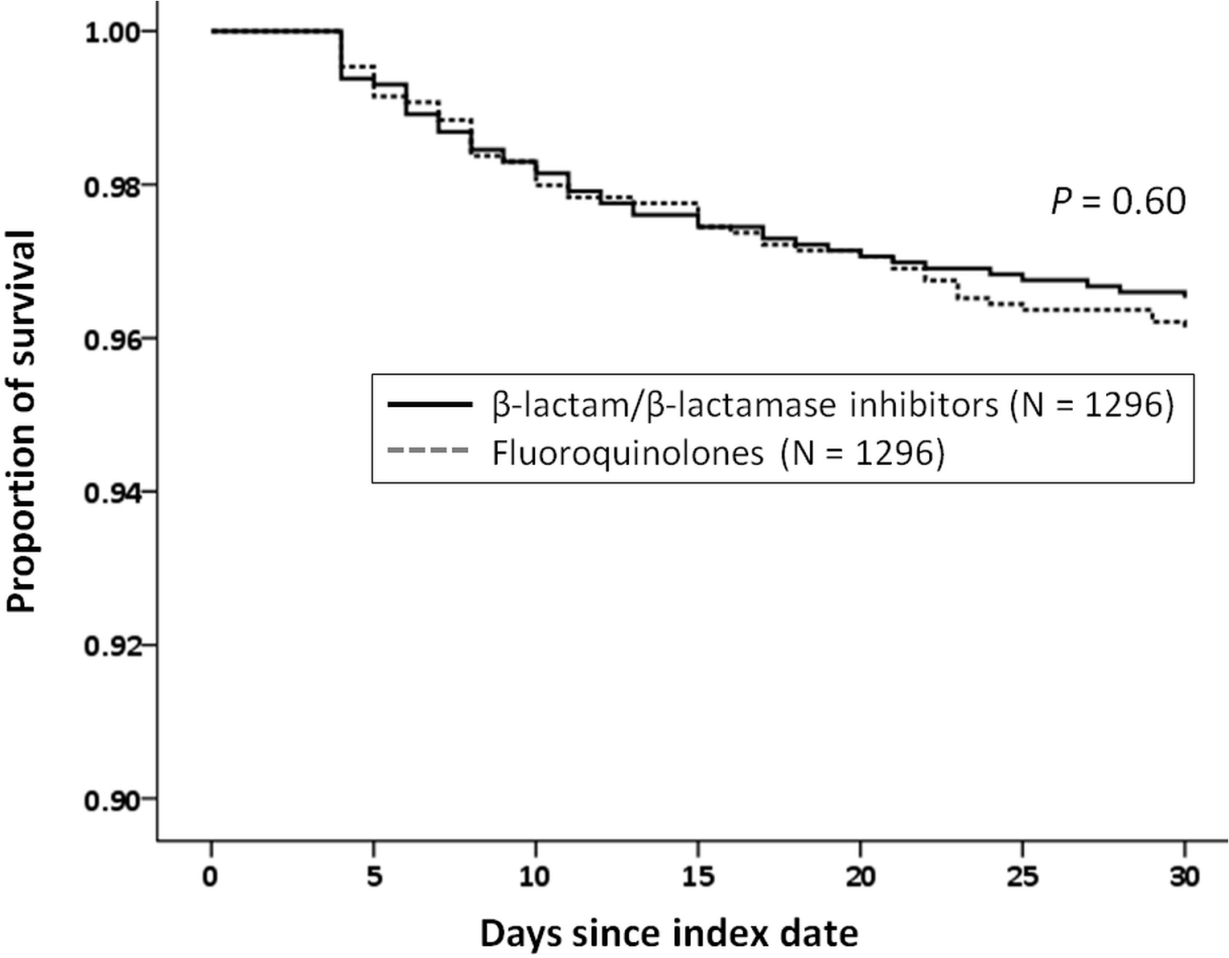
Kaplan-Meier estimates of time to pneumonia/empyema-related hospitalization/ emergency department (ED) visits based on receipt of fluoroquinolones and β-lactam/β-lactamase inhibitors in the matched cohorts.

In the unadjusted cohorts, 194 episodes of subsequent pneumonia or empyema-related hospitalization or ED visits developed in the following 30 days after excluding the events within first 72 hours, which included 53 episodes (3.8%) for fluoroquinolones and 141 episodes (4.1%) for β-lactam/β-lactamase inhibitors (Table 2). The rate of treatment failure within the next 30 days was 29.2% (1,418/4,851), which included 28.4% for fluoroquinolones and 29.6% for β-lactam/β-lactamase inhibitors. The 30-day all-cause mortality rates were both 0.6% in both two treatment groups. After adjusting by propensity score, the percentages of the primary and secondary outcomes are also shown in Table 2. Compared with episodes treated with β-lactam/β-lactamase inhibitors, the adjusted HRs or adjusted ORs of episodes treated with fluoroquinolones were 1.11 (95% CI, 0.74–1.66) for 30-day pneumonia/empyema-related hospitalization or ED visits, 0.86 (95% CI, 0.73–1.02) for 30-day treatment failure, and 1.40 (95% CI, 0.45–4.41) for 30 day all-cause mortality, respectively. No statistically significant differences were observed in the primary and secondary outcomes between the two treatment groups. In episodes with 30-day pneumonia/empyema-related hospitalization or ED visits, the median intervals from the index date to hospitalization or ED visits were 10 days in both fluoroquinolone and β-lactam/β-lactamase inhibitor groups. Kaplan-Meier estimates of time to hospitalization/ED visits are illustrated in Fig 2.

**Table 2:** Compared primary and secondary outcomes of pneumonia in COPD patients receiving fluoroquinolones and β-lactam/β-lactamase inhibitors.

	Unadjusted cohort			Matched cohort		
	n/N^a^ (%)	HRs or ORs (95% CI)	*P*	n/N^a^ (%)	HRs or ORs (95% CI)	*P*
**30-day pneumonia or empyema-related hospitalization or ED visits (without excluding the events within the first 72 hours)**						
β-lactam/β-lactamase inhibitors	214/3456 (6.1)	1.00	-	74/1296 (5.7)	1.00	-
Fluoroquinolones	91/1386 (6.6)	1.07 (0.84–1.36)	0.61	86/1296 (6.6)	1.16 (0.86–1.59)	0.33
**30-day pneumonia or empyema-related hospitalization or ED visits (with excluding the events within the first 72 hours)**						
β-lactam/β-lactamase inhibitors	141/3456 (4.1)	1.00	-	45/1296 (3.5)	1.00	-
Fluoroquinolones	53/1386 (3.8)	0.94 (0.68–1.29)	0.69	50/1296 (3.9)	1.11 (0.74–1.66)	0.60
**30-day treatment failure**						
β-lactam/β-lactamase inhibitors	1025/3456 (29.6)	1.00	-	406/1296 (31.3)	1.00	-
Fluoroquinolones	393/1386 (28.4)	0.94 (0.82–1.08)	0.40	366/1296 (28.2)	0.86 (0.73–1.02)	0.09
**30-day all-cause mortality**						
β-lactam/β-lactamase inhibitors	22/3456 (0.6)	1.00	-	5/1296 (0.4)	1.00	-
Fluoroquinolones	8/1386 (0.6)	0.91 (0.40–2.04)	0.82	7/1296 (0.5)	1.40 (0.45–4.41)	0.56

**Abbreviations:** CI, confidence interval; COPD, chronic obstructive pulmonary disease; ED, emergency department; HR, hazard ratio; OR, odds ratio.

^a^N was the total number of episodes with pneumonia, and n was the number of episodes with primary or secondary outcomes.

To evaluate the impact of the combined use of macrolides in β-lactam/β-lactamase inhibitor group, we compared episodes treated with β-lactam/β-lactamase inhibitors plus macrolides with fluoroquinolones (Table 3). Of 3465 patients in the β-lactam/β-lactamase inhibitor group, 142 (4.1%) patients received β-lactam/β-lactamase inhibitors combined with macrolides. In adjusted cohorts, 139 pairs of episodes were matched. For the events of 30-day pneumonia/empyema-related hospitalization or ED visits, no episode was identified in the fluoroquinolone group. The risk for 30-day pneumonia/empyema-related hospitalization or ED visits, treatment failure, and mortality was not significantly different between episodes treated with fluoroquinolones and β-lactam/β-lactamase inhibitors plus macrolides (All *P* >0.05).

**Table 3:** Compared primary and secondary outcomes of pneumonia in COPD patients receiving fluoroquinolones and β-lactam/β-lactamase inhibitors plus macrolides.

	Unadjusted cohorts			Matched cohorts		
	n/N^a^(%)	HRs or ORs (95% CI)	*P*	n/N^a^ (%)	HRs or ORs (95% CI)	*P*
**30-day pneumonia or empyema-related hospitalization or ED visits (without excluding the events within the first 72 hours)**						
β-lactam/β-lactamase inhibitors plus macrolides	5/142 (3.5)	1.00	-	5/139 (3.6)	1.00	-
Fluoroquinolones	91/1386 (6.5)	1.89 (0.77–4.65)	0.17	1/139 (0.7)	0.20 (0.02–1.70)	0.14
**30-day pneumonia or empyema-related hospitalization or ED visits (with excluding the events within the first 72 hours)**						
β-lactam/β-lactamase inhibitors plus macrolides	4/142 (2.8)	1.00	-	4/139 (2.9)	1.00	-
Fluoroquinolones	53/1386 (3.8)	1.36 (0.49–3.76)	0.69	0/139 (0)	-	-
**30-day treatment failure**						
β-lactam/β-lactamase inhibitors plus macrolides	42/142 (29.6)	1.00	-	41/139 (29.5)	1.00	-
Fluoroquinolones	393/1386 (28.4)	0.94 (0.65–1.38)	0.40	36/139 (25.9)	0.84 (0.49–1.41)	0.50
**30-day all-cause mortality**						
β-lactam/β-lactamase inhibitors plus macrolides	0/142 (0)	1.00	-	0/139 (0)	1.00	-
Fluoroquinolones	8/1386 (0.6)	-	-	2/139 (1.4)	-	-

**Abbreviations:** CI, confidence interval; COPD, chronic obstructive pulmonary disease; ED, emergency department; HR, hazard ratio; OR, odds ratio.

^a^N was the total number of episodes with pneumonia, and n was the number of episodes with primary or secondary outcomes

As for the analysis of the effect of active tuberculosis diagnosis, there were 1255 pairs of episodes matched by propensity score. During the 6-month follow-up, 70 patients (5.6%) and 34 patients (2.7%) were newly diagnosed as tuberculosis in β-lactam/β-lactamase inhibitor and fluoroquinolone groups, respectively. The difference between two groups was significant with an adjusted HR of 0.47 (95% CI, 0.31–0.72; *P* <0.01). The medical expenditures in unadjusted and matched cohorts are shown in Table 4. In the matched cohort, the 30-day total medical costs in fluoroquinolone and β-lactam/β-lactamase inhibitor groups were 528 USD (standard deviation [SD], 1,469 USD) and 455 USD (SD, 1,241 USD), respectively. The 30-day pneumonia-related costs in fluoroquinolone and β-lactam/β-lactamase inhibitor groups were 202 USD (SD, 598 USD) and 155 USD (SD, 622 USD), respectively. Only pneumonia-related costs had a statistically borderline significance between the two treatment groups (*P* = 0.05).

**Table 4:** Total medical cost and pneumonia-related cost in patients with COPD and pneumonia receiving fluoroquinolones and β-lactam/β-lactamase inhibitors.

	Fluoroquinolones	β-lactam/β-lactamase inhibitors	*P*
**30-day total medical cost** ^a^			
Unadjusted cohort	532 ± 1,456	559 ± 1,899	0.59
Matched cohort	528 ± 1,469	455 ± 1,241	0.17
**30-day pneumonia-related cost** ^a^			
Unadjusted cohort	200 ± 584	204 ± 984	0.88
Matched cohort	202 ± 598	155 ± 622	0.05

^a^All costs were calculated in US dollars.

## Discussion

To the best of our knowledge, our study was the first study that attempted to compare the effectiveness of different antibiotic treatment for pneumonia in the COPD outpatient population. In this population-based cohort study, we did not find the statistically significant differences in the effectiveness and medical expenditures between fluoroquinolones and β-lactam/β-lactamase inhibitors. The risk of pneumonia/empyema-related hospitalization or ED visits, treatment failure, and all-cause mortality were all similar between the two treatment groups, whereas the pneumonia-related medical costs were numerically higher in patients receiving fluoroquinolones without the significant difference.

The recommended antibiotics for pneumonia in COPD patients were mainly based on the microbiological distribution. According to the IDSA/ATS guideline for adult CAP, the empirical antibiotics for outpatients with comorbidities, such as chronic lung diseases, should be a respiratory fluoroquinolone or a β-lactam plus a macrolide [8]. Comorbidities increase the likelihood of infection with drug-resistant *S*. *pneumoniae* and enteric Gram-negative bacteria in patients. In the COPD population, the most common causative bacterial organisms of acute exacerbation and pneumonia included *S*. *pneumoniae*, *H*. *influenzae* and *P*. *aeruginosa* [4,7,16]. In Taiwan, the predominant bacteria isolated from patients with AECOPD were *Klebsiella pneumoniae* (19.6%), *P*. *aeruginosa* (16.8%) and *H*. *influenzae* (7.5%), while *S*. *pneumoniae* infection only accounted for 2.4% [17]. In addition, COPD patients with receiving repeated courses of antibiotic treatment might acquire resistant pathogens [18]. The distinctive microbiological pattern and increased antimicrobial resistance prompt a respiratory fluoroquinolone and a β-lactam/β-lactamase inhibitor alone or combined with a macrolide as the mostly frequent preferred regimens [8,18].

Except the IDSA/ATS guideline for pneumonia recommending the choice of antibiotics in the comorbid condition, no other guidelines for pneumonia or COPD offer the specific suggestion for pneumonia in COPD patients with pneumonia [5,7,19,20]. Most studies focused on the clinical characteristics and prognosis of pneumonia in COPD patients [21,22], but the comparisons of effectiveness between different antibiotics are scarce [12]. By contrast, the substantial studies on AECOPD indicated that fluoroquinolones, rather than β-lactam/β-lactamase inhibitors or macrolides, achieved higher bacterial eradication and decreased relapse in the long-term outcomes [9,23–25]. The findings that fluoroquinolones were more effective than β-lactam/β-lactamase inhibitors were considered to be related to higher activity against *H*. *influenzae* and *P*. *aeruginosa* [23]. Our study, the first and largest cohort study on COPD outpatients with pneumonia, evaluated the clinical outcomes of patients receiving fluoroquinolones and β-lactam/β-lactamase inhibitors plus macrolides or not. However, there was no decreased risk of hospitalization or ED visits, treatment failure, and mortality within 30 days after the treatment of fluoroquinolones compared with β-lactam/β-lactamase inhibitors plus macrolides or not. A recent cluster-randomized and crossover trial also showed that neither β-lactam-macrolide combination therapy nor fluoroquinolone monotherapy appeared to be better than β-lactam monotherapy for non-severe CAP [26]. With the low rate of atypical pathogens, this finding reflected that most causative pathogens were adequately covered by the three regimens. Although the microbiological data was deficient in our study, this similar finding may also result from the adequate coverage. In addition, the absence of better outcomes in fluoroquinolones may be associated with the antimicrobial resistance in *H*. *influenzae* and the risk factors for *P*. *aeruginosa* infection. In Taiwan, the resistance rate of *H*. *influenzae* to amoxicillin/clavulanate remained stable (4.2%) during 2004–2010, whereas levofloxacin resistance increased from 2% in 2004 to 24.3% in 2010 [27]. The risk factors for *P*. *aeruginosa* infection in COPD patients included lower forced expiratory volume in the first second, systemic corticosteroid treatment, and admissions in the previous year [4,28]. In our study population, only about 30% and 5% of episodes had long-term corticosteroids use and hospitalization in the past year, which might decrease the possibility of *P*. *aeruginosa* infection.

Taiwan has an intermediate tuberculosis burden of cases, and the annual incidence of tuberculosis was 53/100,000 individuals in 2012 [29]. Empirical use of fluoroquinolones in pneumonia patients causes delayed tuberculosis diagnosis and fluoroquinolones resistance of *Mycobacterium tuberculosis* [30]. In present study, the percentage of newly diagnosed tuberculosis within 6 months was higher in the β-lactam/β-lactamase inhibitor group instead of the fluoroquinolone group. Our clinical practice with prudent to avoid the empirical use or shorten treatment course of fluoroquinolones in patients at high risk for tuberculosis may attribute the result [30]. As for medical economics, past few studies suggested that patients treated with fluoroquinolones had the lower medical expenditures compared with those treated with β-lactam/β-lactamase inhibitors plus macrolides [31,32]. However, our findings show the numerically higher pneumonia-related medical costs in patients receiving fluoroquinolones. All the aforementioned clinical and economic outcomes should be carefully balanced before prescription.

Our study has several limitations. First, the diagnoses of COPD and pneumonia, based on ICD-9-CM, may be indistinct from other respiratory diseases due to the similar symptoms. To increase the validity of the diagnoses, we used more strict criteria, such as at least 2 outpatient diagnoses of COPD or pneumonia episodes plus a confirmatory diagnostic procedure. In addition, more than 95% of episodes (97.2% in both the unadjusted and adjusted cohorts) had used at least one COPD-related medication (i.e. inhaled short-acting and long-acting β2-agonists, inhaled anticholinergics, inhaled corticosteroids and theophyllines) during the study period. Second, the information on severity of spirometric abnormality, pneumonia, laboratory data, and imaging findings were not available in a claims database. However, we collected most obtainable underlying diseases and past history of medication and healthcare utilization. The propensity score analysis was also performed to increase comparability among treatment groups. Third, the outcomes of bacterial eradication or resistance rate were impossible to assess because the etiology and their drug susceptibility testing of pneumonia were unavailable in the database. Lastly, our study was conducted in Taiwan and underpowered due to the low number of outcome events. Albeit the large database, the results may not be generalized to other populations or geographic locations.

In conclusion, fluoroquinolones and β-lactam/β-lactamase inhibitors were associated with similar clinical outcomes and medical expenditures for pneumonia among COPD outpatients. Further prospective comparative studies to determine the most cost-effective antibiotic treatment are warranted.
